# Suspected Central Adrenal Insufficiency in a Patient with Phosphomannomutase 2–Congenital Disorder of Glycosylation

**DOI:** 10.1210/jcemcr/luaf237

**Published:** 2025-10-10

**Authors:** Sofie-Louise Feentved Ødum, Sabine W Grønborg, Katharina M Main, Marianne Klose

**Affiliations:** Department of Nephrology and Endocrinology, Copenhagen University Hospital, Rigshospitalet, DK-2100 Copenhagen, Denmark; Center for Inherited Metabolic Diseases, Department of Pediatrics and Adolescent Medicine and Department of Clinical Genetics, Copenhagen University Hospital, Rigshospitalet, DK-2100 Copenhagen, Denmark; Department of Growth and Reproduction, Copenhagen University Hospital, Rigshospitalet, DK-2100 Copenhagen, Denmark; International Center for Research and Research Training in Endocrine Disruption of Male Reproduction and Child Health (EDMaRC), Copenhagen University Hospital, Rigshospitalet, DK-2100 Copenhagen, Denmark; Department of Clinical Medicine, Faculty of Health and Medical Sciences, University of Copenhagen, DK-2100 Copenhagen, Denmark; Department of Nephrology and Endocrinology, Copenhagen University Hospital, Rigshospitalet, DK-2100 Copenhagen, Denmark

**Keywords:** PMM2-CDG, CBG, adrenal insufficiency, glycosylation

## Abstract

Phosphomannomutase 2–congenital disorder of glycosylation (PMM2-CDG) is a hereditary defect causing hypoglycosylation of N-linked glycoproteins. It was recently suggested that patients with PMM2-CDG may have central adrenal insufficiency. We present an 18-year-old male with PMM2-CDG, whose initial screening suggested adrenal insufficiency. The patient displayed a morning plasma cortisol of 57 nmol/L (2.07 μg/dL) (reference range, 133-537 nmol/L [4.82-19.46 μg/dL]), a 30-minute ACTH-stimulated cortisol of 165 nmol/L (5.98 μg/dL), and a low-normal ACTH. The patient never experienced obvious symptoms of adrenal insufficiency nor clinical improvement after transient introduction of hydrocortisone. Additional assessment was therefore conducted, including cortisol binding globulin, which was markedly low (253 mmol/L (14.7 mg/L) (reference range, 750-2500 mmol/L [43.7-93.1 mg/L])). Subsequently, 8 Am salivary cortisol measured 24 hours after last hydrocortisone ingestion was normal-to-slightly elevated (38 nmol/L (1.38 μg/dL) (reference range, 2.48-29 nmol/L [0.09-1.05 μg/dL])). In conclusion, we present a patient with PMM2-CDG who, upon measurement of plasma cortisol, displayed “biochemical” adrenal insufficiency, although with low cortisol binding globulin and normal morning salivary free cortisol levels. This case illustrates the caveats of total cortisol for the diagnosis of adrenal insufficiency in patients with PMM2-CDG and highlights the potential impact of N-linked hypoglycosylation on endocrine evaluation.

## Introduction

The most common form of congenital disorders of glycosylation (CDG) is phosphomannomutase 2-CDG (PMM2-CDG) [1-3]. Reduced PMM2 enzyme activity impairs N-glycosylation of glycosylated proteins, leading to a spectrum of clinical presentations, from severe neurological-multiorgan disease to an isolated neurological syndrome [3]. Infants with multisystem presentation have developmental delay with axial hypotonia and hyporeflexia, strabismus, and failure to thrive. Typically, patients have abnormal fat distribution with suprapubic or supragluteal fat pads and inverted nipples. Severe organ complications, including hepatopathy, pericardial effusion, cardiomyopathy, enteropathy, hypoalbuminemia, ascites, and increased infection risk, can be life threatening [4]. Other patients have neurological involvement with developmental delay, intellectual disability, cerebellar ataxia, epilepsy, and stroke-like episodes. Patients can develop neuropathy and retinitis pigmentosa, which can be accompanied by milder visceral symptoms, infections, and skeletal manifestations like scoliosis and kyphosis [1, 2].

Reduction of PMM2 enzyme activity impairs conversion of mannose-6-phosphate to mannose-1-phosphate, resulting in lack of guanosine diphosphate-mannose, which is essential for N-linked glycoprotein formation, thus causing hypoglycosylation [1]. N-linked glycosylation influences stability, binding, and ligand-specificity of polypeptide hormones, and the corresponding hormone receptors, binding proteins, and downstream pathways [1]. A reduction in the mannose-6-phosphate/mannose-1-phosphate conversion may therefore—theoretically—affect all endocrine axes with N-glycosylated hormones, such as GH, LH, FSH [5], and glycosylated hormone binding proteins (such as thyroxine binding globulin [TBG] and cortisol binding globulin [CBG]) [3].

Recently, Čechová et al [6]. published a study suggesting that patients with PMM2-CDG are at risk of developing central adrenal insufficiency. They reported an increased frequency of adrenal insufficiency in a cohort of 43 patients with PMM2-CDG, where 11 patients had morning (7:45-11:50 Am) plasma cortisol levels lower than 138 nmol/L (5.0 μg/dL) [6]. The authors concluded that all patients with PMM2-CDG should be screened annually, prompting the focus on adrenal insufficiency in our case.

## Case Presentation

An 18-year-old male with PMM2-CDG (genotype: *PMM2* c.357C>A, p.(Phe119Leu); c.422G>A, p.(Arg131His)) [7] was referred to our Department of Endocrinology on suspicion of adrenal insufficiency due to morning cortisol concentration of 57 nmol/L (2.07 μg/dL)(reference range, 133-537 nmol/L [4.82-19.46 μg/dL]) on routine laboratory testing.

The patient was diagnosed with PMM2-CDG at the age of 2 years, following tonic-clonic seizures at 18 months. Delayed psychomotor development and dysmorphia with inverted nipples prompted transferrin isoelectric focusing, revealing a pathological type-I transferrin pattern; PMM2-CDG diagnosis was confirmed by genetic sequencing. Throughout his childhood, he was frequently hospitalized for airway infection and multifocal epilepsy. In relation to epileptic seizures and head trauma, he experienced several stroke-like episodes with transient hemiparesis. At age 18 years, he is intellectually disabled, nonverbal but communicates nonverbally, recognizes familiar people, and is fully dependent on continuous care. He is wheelchair-bound, has reduced vision due to myopia and tapetoretinal dystrophy, and has skeletal issues typical for PMM2-CDG, including pectus carinatum and kyphosis. He requires percutaneous gastrostomy for feeding. He has intractable epilepsy and is medicated with lacosamide, levetiracetam, and clobazam.

The patient was unable to cooperate physically or verbally; thus, statements from parents and his health care team were used to determine the clinical picture of the patient. He had no history of symptoms suggestive of adrenal insufficiency, neither in general nor during hospitalization for respiratory infections or seizures, or disproportionately illness during past infections.

## Diagnostic Assessment

At referral, the patient had an 8 Am plasma cortisol of 57 nmol/L (2.07 μg/dL) and ACTH of 1.7 pmol/L (7.73 pg/mL) (reference range, 1.3-16.7 pmol/L [5.91-75.91 pg/mL]), indicative of central adrenal insufficiency. The patient had never been treated with systemic or topic glucocorticoids before assessment. Subsequent assessments included a 250-μg ACTH stimulation test and full anterior pituitary assessment (Table 1). Adrenal axis assessment confirmed low baseline morning cortisol of 38 nmol/L (1.38 μg/dL) and a low 30-minute ACTH-stimulated cortisol of 165 nmol/L (5.98 μg/dL). Similarly, thyroid axis assessment showed low total and free thyroxine (T4) with mid-normal TSH and low TBG, whereas gonadal assessment showed high-normal total testosterone with elevated gonadotrophin concentration. Because of the lack of obvious symptoms of adrenal insufficiency, including rapid and uneventful recovery from infectious diseases, the diagnosis of adrenal insufficiency was questioned. The diagnostic setup was supplemented by liquid chromatography-tandem mass spectrometry analysis of cortisol, measurement of CBG as well as salivary free cortisol. Cortisol measured by liquid chromatography-tandem mass spectrometry confirmed the plasma-cortisol concentrations measured by RocheCortII. The CBG concentration was below the lower reference limit (253 nmol/L [14.7 mg/L]) (reference range, 750-1600 nmol/L [43.7-93.1 mg/L]). Salivary cortisol was measured in the morning before and after administration of hydrocortisone, as well as in the afternoon before and after the second hydrocortisone dose, showing a normal morning salivary cortisol concentration with an expected increase following hydrocortisone intake (Table 1).

**Table 1.** Laboratory results

Test	Results	Reference range	Method
**Plasma cortisol**	**08:40 Am:** **57 nmol/L (2.07 μg/dL)**	Morning: 133-537 nmol/L (4.82 -19.46 μg/dL)	Competitive electrochemiluminescence immunoassay Roche diagnostics, Cortisol II
**Synacthen test**			
** 0-minute cortisol**	**38 nmol/L (1.38 μg/dL)**		Competitive electrochemiluminescence immunoassay Roche diagnostics, Cortisol II
** 30-minute cortisol**	**165 nmol/L (5.98 μg/dL)**	Cutoff 420 nmol/L (15.22 μg/dL)	
**ACTH**	1.7 pmol/L (7.73 pg/mL)	1.3-16.7 pmol/L (5.91-75.91 pg/mL)	Sandwich electrochemiluminescence immunoassay, Cobas, Elecsys ACTH
**CBG**	**253 nmol/L (14.7 mg/L)**	750-1600 nmol/L (43.7-93.1 mg/L)	Radioimmunoassay (BioSource inc. Worcester, MA, USA)
**Albumin**	**29 g/L (2.90 g/dL)**	36-48 g/L (3.6-4.8 g/dL)	Colorimetric method slide, VITROS chemistry products ALB Slides REF 819 6057
**Salivary cortisol**			
** 8:00 Am** **before HC**	**38 nmol/L (1.38 μg/dL)**	06:00-11:00: 2.48-29 nmol/L (0.09-1.05 μg/dL)	Chemiluminescence immunoassay, IDS-iSYS salivary cortisol package insert IS-4900
** 8:30 Am** **after HC**	**>60 nmol/L (>2.17 μg/dL)**		
** 4:30 Pm** **before HC**	6 nmol/L (0.22 μg/dL)	20:00-00:35: 0.55-9.4 nmol/L (0.02-0.34 μg/dL)	
** 7:00 Pm 2 hours after HC**	**30 nmol/L (1.09 μg/dL)**		
**TSH**	1.85 mIU/L (1.85 **μ**IU/mL)	1.06-5.80 mIU/L (1.06-5.80 μIU/mL)	Sandwich electrochemiluminescence immunoassay, Cobas TSH Thyrotropin
**Total T3**	1.5 nmol/L (97.4 ng/dL)	1.0-2,6 nmol/L (64.94-168.83 ng/dL)	Competitive electrochemiluminescence immunoassay, Cobas T3 triiodothyronine
**Total T4**	**46 nmol/L (3.57 μg/dL)**	70-140 nmol/L (5.44-10.88 μg/dL)	Competitive electrochemiluminescence immunoassay, Cobas T4 Thyroxin
**Free T4**	**10.8 pmol/L (0.8 ng/dL)**	12.0-22.0 pmol/L (0.9-1.7 ng/dL)	Competitive electrochemiluminescence immunoassay, Cobas FT4 (free thyroxine)
**TBG**	**123 nmol/L (7.2 μg/mL)**	259-574 nmol/L (15.2-33.6 μg/mL)	Competitive electrochemiluminescence immunoassay, WHO International Standard 88/638
**Testosterone**	**35 nmol/L (1009.5 ng/dL)**	8.6-29 nmol/L (248.0-836.4 ng/dL)	Competitive electrochemiluminescence immunoassay, Cobas Testosterone II
**LH**	**10.1 IU/L (10.1 mIU/mL)**	1.7-8.6 IU/L (1.7-8.6 mIU/mL)	Sandwich electrochemiluminescence immunoassay, Cobas LH luteinizing hormone
**FSH**	**22.8 IU/L (22.8 mIU/mL)**	1.5-12.9 IU/L (1.5-12.9 mIU/mL)	Sandwich electrochemiluminescence immunoassay, Cobas FSH follicle stimulating hormone
**SHBG**	**62.0 nmol/L (6.97 μg/mL)**	15.8-55.5 nmol/L (1.78-6.27 μg/mL)	Sandwich electrochemiluminescence immunoassay, Roche SHBG
**Prolactin**	107 mIU/L (107 **μ**IU/mL	69-266 mIU/L (69-266 μIU/mL)	Immunofluorometric assay, B.R.A.H.M.S prolactin KRYPTOR
**IGF-1**	210 ug/L (210 ng/mL)	Gender and age adjusted −1.51 SD	Chemiluminescent Immunoassay, IDS iSYS
**Sodium**	139 mmol/L (139 mEq/L)	135-147 mmol/L (135-147 mEq/L)	Ion selective electrode measuring—potentiometry
**Potassium**	3.4 mmol/L (3.4 mEq/L)	3.3-4.3 nmol/L (3.3-4.3 mEq/L)	Ion selective electrode measuring—potentiometry

Laboratory results outside of reference ranges are shown in bold font.

Abbreviations: CBG, cortisol binding globulin; HC, hydrocortisone; TBG, thyroxine-binding globulin; T4, thyroxine; T3, triiodothyronine.

## Treatment

The patient was initially prescribed oral hydrocortisone 10 Am and 5 mg at noon, which was discontinued after ruling out the diagnosis. No amelioration of the patient’s general well-being was observed during treatment.

## Outcome and Follow-up

Based on the absence of clinical signs and symptoms and supportive biochemical findings, the patient was considered to have normal hypothalamic–pituitary–adrenal function. Hydrocortisone treatment was therefore withheld, without eliciting any clinical deterioration. Consequently, the patient and caretakers were spared the significant burden of managing a diagnosis of adrenal insufficiency, which often includes difficult decisions about stress doses, emergency hospitalizations, and complex long-term care decisions.

## Discussion

**Figure 1.**
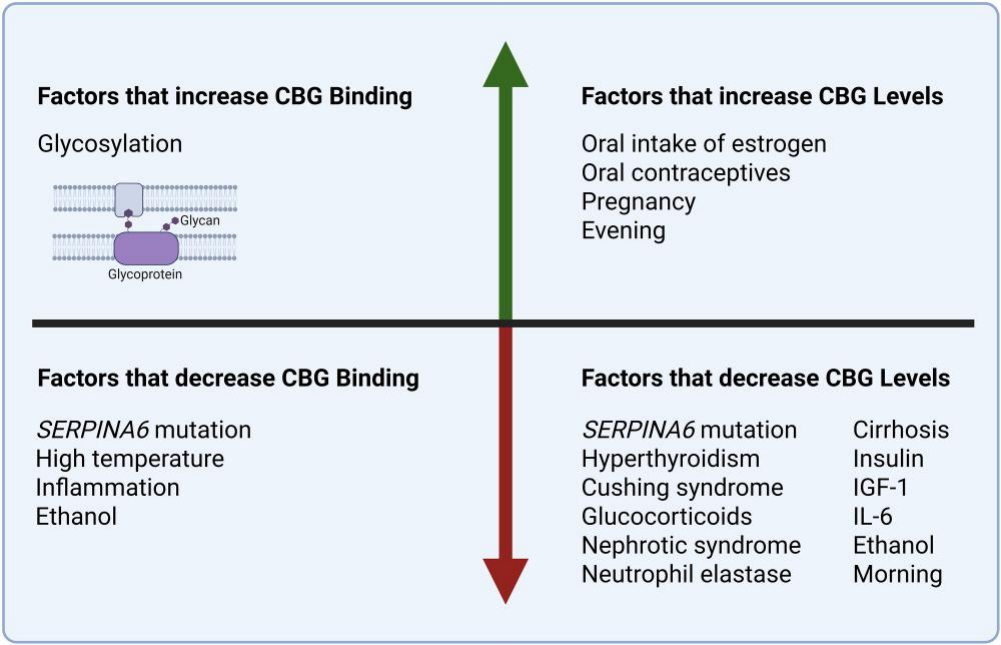
Factors affecting binding affinity for cortisol and serum concentration of CBG. Created in BioRender. Feentved, S. (2025) https://BioRender.com/c10d351. CBG, cortisol binding globulin; IL-6, interleukin 6.

This patient with PMM2-CDG presented with biochemical suspicion of central adrenal insufficiency. Further examination showed abnormal low CBG, and normal levels of free cortisol, and the patient was finally considered to have normal hypothalamic–pituitary–adrenal function. PMM2-CDG syndrome is a disorder of N-linked protein glycosylation characterized by protein and lipid hypoglycosylation [1, 2]. CBG is a heavily glycosylated protein [8-11]. It is characterized by 6 N-linked glycosylation sites, and up to 30% of CBG consists of glycans [8, 9]. CBG is responsible for binding and transporting cortisol [12, 13]. The amount of active free cortisol is strictly regulated via a balanced, dynamic equilibrium between free cortisol (<10%), cortisol bound to high-affinity CBG (80%), and to a lesser extend to albumin with low affinity (10%) [13-15]. This equilibrium is influenced by multiple factors, as summarized in Fig. 1. Glycosylation of CBG increases the cortisol binding-capacity, whereas fever, inflammation, alcohol, and *SERPINA6* mutations decrease the cortisol-biding capacity of CBG. Factors that change CBG levels also impact this equilibrium [16]. Contraceptives, for instance, increase CBG levels and thereby total cortisol, whereas other factors (eg, glucocorticoids, Cushing syndrome, hyperthyroidism, alcohol, certain *SERPINA6* mutations) are associated with decreased CBG levels and lower total cortisol concentrations (Fig. 1) [16, 17].

Glycosylation of proteins modulates and mediates a wide variety of functions in physiological and pathophysiological states [18]. Glycosylation impacts protein folding and conformation, stability in plasma, as well as interaction with proteases, receptors, and carbohydrate-binding proteins. N-glycosylation was shown to be crucial for a high steroid-binding affinity of CBG compared with unglycosylated recombinant CBG [9]. Thus, glycosylation increased binding affinity 9-fold for high-affinity CBG and 2.5-fold for low affinity CBG [8, 9, 11, 19]. Furthermore, site-specific N-glycosylation was shown to differentially affect the binding affinity of CBG [8, 9, 19]. Disruption of the Asn238 N-glycosylation site has been associated with a significant loss of cortisol-binding capacity [8]. Similarly, the oligosaccharide structures of heterodimeric glycoprotein hormones, such as FSH, play an important role in the biosynthesis, secretion, metabolism, and regulation of FSH potency. Thus, alternative glycosylation of hormones such as FSH may affect the metabolic clearance and in vivo biopotency of the hormone [5]. Whether this could explain the discrepant finding of compensated hypergonadotropic hypogonadism, or whether patients with PMM2-CDG have primary gonadal dysfunction remains unknown. Notably, SHBG is less glycosylated as compared to CBG and TBG, which might explain the discrepancy compared to the adrenal and thyroid axis, though this remains speculative [9, 20].

In the present case, no medication with potential impact on the adrenal axis was taken at or before the time of endocrine testing. The patient had no signs of inflammation and fever that could modify cortisol binding capacity of CBG. This suggests that such confounding factors do not explain the observed discrepancy between markedly low total plasma cortisol and normal salivary free cortisol.

Recent data support the high sensitivity and specificity of early morning salivary cortisol for ruling out adrenal insufficiency, with a cutoff well below the salivary cortisol of 38 nmol/L observed in our patient [21]. We were therefore confident in excluding the diagnosis. Salivary samples collected before and after ACTH stimulation were attempted, but the salivary volume was insufficient for analysis. Assessment of 24-hour urinary free cortisol assessment was also considered but not performed, as this would require urinary catheterization, which was deemed unnecessarily invasive. A corticotropin-releasing hormone test could have provided valuable diagnostic insight, but unavailable due to the discontinuation of CRH production by the global manufacturer.

The decreased level of CBG could explain the decreased total plasma cortisol. It remains unknown whether the decrease in total cortisol is solely caused by the lowered CBG. A reference for the total cortisol/CBG index as a surrogate measure of free cortisol has not been established to exclude adrenal insufficiency and may be inaccurate in patients with CBG affinity changes [22].

Little is known about the clinical relevance of low CBG. CBG belongs to the SERPIN protein family, which encodes various highly glycosylated proteins. Certain mutations in the SERPIN family genes encoding CBG (*SERPINA6)* are associated with low or noncirculating CBG, whereas some polymorphisms only affect the hormone-binding structure are associated with normal serum levels of CBG [23]. Importantly, *SERPINA6* mutations are associated with normal free cortisol levels and ACTH despite reduced total cortisol [16]. Yet, patients with these variants may still experience fatigue and hypotension, not directly attributed to adrenal insufficiency per se [23-27].

The SERPIN genes encode various N-glycosylated proteins including CBG and TBG (Table 2). Serpins are conformationally labile, and many disease-linked mutations result in misfolding or in pathogenic inactive polymers. As with CBG, TBG contains approximately 20% of carbohydrates by weight. Carbohydrates affect the half-life of TBG in serum and reduce TBG immunoreactivity, T4-binding activity, and stability [28, 29], which may explain the biochemical alterations in the thyroid axis observed in our case. Mutations in the *SERPINA7* gene induce low TBG and low-normal total T4 but with a lack of clinical symptoms and normal free T4 [30].

**Table 2.** Section of relevant SERPIN genes

SERPIN family genes	Names	Function of the protein	Clinical features with deficiency
*SERPINA1*	Alpha 1-antitrypsin	Inhibition of neutrophil elastase	Emphysema and cirrhosis
*SERPINA3*	Antichymotrypsin	Inhibition of cathepsin g	Emphysema
*SERPINA5*	Protein C-inhibitor (PAI-3)	Inhibition of the active form of protein C	Angioedema
*SERPINA6*	Cortisol-binding globulin	Binding of free cortisol	Chronic fatigue and affected blood pressure
*SERPINA7*	Thyroxine-binding globulin	Binding of free thyroxine	Thyroxine-binding globulin deficiency

Future studies are warranted to explore whether SERPIN proteins, such as CBG and TBG, are structurally modified in PMM2-CDG and if such alterations affect their physiological functions. Such modifications could potentially explain the biochemical suspicion of adrenal insufficiency and hypothyroidism.

In conclusion, this patient with PMM2-CDG showed biochemical findings suggestive of central adrenal insufficiency but without clinical symptoms and with normal free cortisol concentrations. Given that disorders of glycosylation may reduce hormone-binding globulin concentrations and affinity, caution is warranted in evaluating adrenal function in patients with PMM2-CDG, to avoid a false-positive diagnosis and unnecessary, potentially harmful treatment.

## Learning Points

- Low cortisol levels in patients with PMM2-CDG may be caused by low CBG and might not necessarily require substitution therapy.
- Evaluation of adrenal function in patients with PMM2-CDG should include measurement of CBG and free cortisol if low cortisol levels are observed in routine investigations.
- The glycosylation defect in PMM2-CDG syndrome may decrease cortisol affinity of CBG ensuring appropriate tissue free cortisol.

## Contributors

All authors made contributions to authorship. S.G., M.K., and K.M. were involved in the diagnosis and management of this patient. S.L.F.O. and M.K. undertook data acquisition. All authors were involved in the interpretation of data and drafting and reviewing the manuscript. All authors have approved and are accountable for the final manuscript.

## Data Availability

Data sharing is not applicable to this article as no datasets were generated or analyzed during the current study.

## References

[1] Chang IJ, He M, Lam CT. Congenital disorders of glycosylation. Ann Transl Med. 2018;6(24):477‐477. 30740408 10.21037/atm.2018.10.45 PMC6331365

[2] Sparks SE, Krasnewich DM. PMM2-CDG. GeneReviews® 2021. Accessed May 16, 2024. https://www.ncbi.nlm.nih.gov/books/NBK1110/

[3] Altassan R, Péanne R, Jaeken J, et al International clinical guidelines for the management of phosphomannomutase 2-congenital disorders of glycosylation: diagnosis, treatment and follow up. J Inherit Metab Dis. 2019;42(1):5‐28. 30740725 10.1002/jimd.12024

[4] Grünewald S . The clinical spectrum of phosphomannomutase 2 deficiency (CDG-Ia). Biochim Biophys Acta. 2009;1792(9):827‐834. 19272306 10.1016/j.bbadis.2009.01.003

[5] Ulloa-Aguirre A, Maldonado A, Damián-Matsumura P, Timossi C. Endocrine regulation of gonadotropin glycosylation. Arch Med Res. 2001;32(6):520‐532. 11750727 10.1016/s0188-4409(01)00319-8

[6] Čechová A, Honzík T, Edmondson AC, et al Should patients with phosphomannomutase 2-CDG (PMM2-CDG) be screened for adrenal insufficiency? Mol Genet Metab. 2021;133(4):397‐399. 34140212 10.1016/j.ymgme.2021.06.003 PMC8754259

[7] Vaes L, Rymen D, Cassiman D, et al Genotype–phenotype correlations in PMM2-CDG. Genes (Basel). 2021;12(11):1658. 34828263 10.3390/genes12111658 PMC8620515

[8] Sumer-Bayraktar Z, Kolarich D, Campbell MP, Ali S, Packer NH, Thaysen-Andersen M. N-glycans modulate the function of human corticosteroid-binding globulin. Mol Cell Proteomics. 2011;10(8):M111.009100. 10.1074/mcp.M111.009100 PMC3149095 21558494

[9] Simard M, Underhill C, Hammond GL. Functional implications of corticosteroid-binding globulin N-glycosylation. J Mol Endocrinol. 2018;60(2):71‐84. 29273683 10.1530/JME-17-0234 PMC5793714

[10] Lee JH, Meyer EJ, Nenke MA, Falhammar H, Torpy DJ. Corticosteroid-binding globulin (CBG): spatiotemporal distribution of cortisol in sepsis. Trends Endocrinol Metab. 2023;34(3):181‐190. 36681594 10.1016/j.tem.2023.01.002

[11] Lee JH, Meyer EJ, Nenke MA, Lightman SL, Torpy DJ. Cortisol, stress, and disease—bidirectional associations; role for corticosteroid-binding globulin? J Clin Endocrinol Metab. 2024;109(9):2161‐2172. 38941154 10.1210/clinem/dgae412

[12] Strel’chyonok OA, Avvakumov GV. Interaction of human CBG with cell membranes. J Steroid Biochem Mol Biol. 1991;40(4-6):795‐803. 1659892 10.1016/0960-0760(91)90305-o

[13] Lewis JG, Lewis MG, Elder PA. An enzyme-linked immunosorbent assay for corticosteroid-binding globulin using monoclonal and polyclonal antibodies: decline in CBG following synthetic ACTH. Clin Chim Acta. 2003;328(1-2):121‐128. 12559607 10.1016/s0009-8981(02)00417-5

[14] El-Farhan N, Rees DA, Evans C. Measuring cortisol in serum, urine and saliva—are our assays good enough? Ann Clin Biochem. 2017;54(3):308‐322. 28068807 10.1177/0004563216687335

[15] Choi MH . Clinical and technical aspects in free cortisol measurement. Endocrinol Metab. 2022;37(4):599‐607. 10.3803/EnM.2022.1549 PMC9449105 35982612

[16] Verbeeten KC, Ahmet AH. The role of corticosteroid-binding globulin in the evaluation of adrenal insufficiency. J Pediatr Endocrinol Metab. 2018;31(2):107‐115. 29194043 10.1515/jpem-2017-0270

[17] Klose MC, Lange M, Rasmussen AK, et al Factors influencing the adrenocorticotropin test: role of contemporary cortisol assays, body composition, and oral contraceptive agents. J Clin Endocrinol Metab. 2007;92(4):1326‐1333. 17244781 10.1210/jc.2006-1791

[18] Reily C, Stewart TJ, Renfrow MB, Novak J. Glycosylation in health and disease. Nat Rev Nephrol. 2019;15(6):346‐366. 30858582 10.1038/s41581-019-0129-4 PMC6590709

[19] Simard M, Hill LA, Lewis JG, Hammond GL. Naturally occurring mutations of human corticosteroid-binding globulin. J Clin Endocrinol Metab. 2015;100(1):E129‐E139. 25322275 10.1210/jc.2014-3130

[20] Lewis JG, Elder PA. Glycosylation may influence sex hormone-binding globulin measurements. Clin Chim Acta. 2020;509:95‐100. 32531254 10.1016/j.cca.2020.06.016

[21] Debono M, Elder CJ, Lewis J, et al Home waking salivary cortisone to screen for adrenal insufficiency. NEJM Evid. 2023;2(2):EVIDoa2200182. 38320034 10.1056/EVIDoa2200182

[22] Fede G, Spadaro L, Tomaselli T, et al Comparison of total cortisol, free cortisol, and surrogate markers of free cortisol in diagnosis of adrenal insufficiency in patients with stable cirrhosis. Clin Gastroenterol Hepatol. 2014;12(3):504‐512.e8. 23978347 10.1016/j.cgh.2013.08.028

[23] Janciauskiene S, Lechowicz U, Pelc M, Olejnicka B, Chorostowska-Wynimko J. Diagnostic and therapeutic value of human serpin family proteins. Biomed Pharmacother. 2024;175:116618. 38678961 10.1016/j.biopha.2024.116618

[24] Law RHP, Zhang Q, McGowan S, et al An overview of the serpin superfamily. Genome Biol. 2006;7(5):216. 16737556 10.1186/gb-2006-7-5-216 PMC1779521

[25] Torpy DJ, Bachmann AW, Gartside M, et al Association between chronic fatigue syndrome and the corticosteroid-binding globulin gene ALA SER224 polymorphism. Endocr Res. 2004;30(3):417‐429. 15554358 10.1081/erc-200035599

[26] Torpy DJ, Bachmann AW, Grice JE, et al Familial corticosteroid-binding globulin deficiency due to a novel null mutation: association with fatigue and relative hypotension. J Clin Endocrinol Metab. 2001;86(8):3692‐3700. 11502797 10.1210/jcem.86.8.7724

[27] Meyer EJ, Nenke MA, Rankin W, Lewis JG, Torpy DJ. Corticosteroid-binding globulin: a review of basic and clinical advances. Horm Metab Res. 2016;48((06|6)):359‐371. 27214312 10.1055/s-0042-108071

[28] Mori Y, Seino S, Takeda K, et al A mutation causing reduced biological activity and stability of thyroxine-binding globulin probably as a result of abnormal glycosylation of the molecule. Mol Endocrinol. 1989;3(3):575‐579. 2501669 10.1210/mend-3-3-575

[29] Murata Y, Magner JA, Refetoff S, Refetoff S. The role of glycosylation in the molecular conformation and secretion of thyroxine-binding globulin. Endocrinology. 1986;118(4):1614‐1621. 3081330 10.1210/endo-118-4-1614

[30] Mimoto MS, Refetoff S. Clinical recognition and evaluation of patients with inherited serum thyroid hormone binding protein mutations. J Endocrinol Invest. 2019;43(1):31. 31352644 10.1007/s40618-019-01084-9 PMC6954308

